# Muometric positioning system (μPS) with cosmic muons as a new underwater and underground positioning technique

**DOI:** 10.1038/s41598-020-75843-7

**Published:** 2020-11-03

**Authors:** Hiroyuki K. M. Tanaka

**Affiliations:** 1 Earthquake Research Institute, The University of Tokyo, 1-1-1 Yayoi, Bunkyo, Tokyo, 113-0032 Japan; 2 International Muography Research Organization (MUOGRAPHIX), The University of Tokyo, 1-1-1 Yayoi, Bunkyo, Tokyo, 113-0032 Japan

**Keywords:** Geophysics, Experimental particle physics

## Abstract

Thus far, underwater and underground positioning techniques have been limited to those using classical waves (sound waves, electromagnetic waves or their combination). However, the positioning accuracy is strongly affected by the conditions of media they propagate (temperature, salinity, density, elastic constants, opacity, etc.). In this work, we developed a precise and entirely new three-dimensional positioning technique with cosmic muons. This muonic technique is totally unaffected by the media condition and can be universally implemented anywhere on the globe without a signal transmitter. Results of our laboratory-based experiments and simulations showed that, for example, plate-tectonics-driven seafloor motion and magma-driven seamount deformation can be detected with the μPS.

## Introduction

Precise underwater and underground positionings are required for submarine^1–3^ or submerged^4,5^ volcano monitoring, slow slip observations^6^, coseismic displacement measurements^7–9^, and multiple engineering purposes^10–12^. These works have utilized the techniques of a combination of GPS and the acoustic positioning system, ocean bottom pressure gauges, autonomous-underwater-vehicles-based sonar bathymetry, Wi-Fi location technology, radio-frequency identification technology, strainmetry, and inertial navigation.

The acoustic positioning system has been widely used for the purpose of underwater positioning. The technique measures travel times of acoustic signals between the sea level transducer and an array of transponders on the seafloor^13,14^. Fluctuations of the acoustic wave velocity in seawater limits this technique's accuracy and dependability^15,16^. Compared to air, the fluctuations of the oceanic environment, e.g., variations in salinity, temperature, or internal density waves in the upper ocean, provide a considerably less predictable propagation medium for sound. In particular, at shallow depths of seawater near coastal areas, sound propagation is strongly affected by solar radiation, seasonal cycles, mixing of the water due to sea currents, and the presence of rivers or waste waters^17^. Ocean bottom pressure gauging is also a popular technique for detecting the seafloor displacement. It enables us to measure seawater pressure with a precision of sub-centimeter water equivalent^18^, however, the technique has an intrinsic drift error, and it only provides the vertical information.

Cosmic-ray muons that have a rest mass of 105 MeV are in general strongly relativistic particle, traveling almost at the speed of light through any kind of material and their speed is not affected by the media condition they travel as long as their energies are within the relativistic region. Cosmic-ray muons (or atmospheric muons) are ubiquitous and universal. They are produced in the air via the collision between primary cosmic rays (mostly galactic cosmic rays (GCRs)) and nuclei in the atmosphere. The produced secondary mesons (pions and kaons) by this reaction subsequently decay in to muons. GCRs are deflected during their propagation in the galaxy, and lose their initial directional information before arriving the Earth. On the other hand, due to the different meson’s mean free paths in the atmosphere, the muon flux varies for different arriving zenith angles. As a consequence, the vertical muon flux is higher than the horizontal flux. The vertical muon flux is ~ 10^2^ m^−2^ s^−1^sr^−1^, but this flux is reduced to ~ 10^–2^ m^−2^ s^−1^sr^−1^ at a depth of 10^3^ m water equivalent (m.w.e.)^19^. The muons that can penetrate water with thicknesses more than 1 m are relativistic (300-MeV, 2-GeV, 30 GeV and 300-GeV muons respectively have continuous slowing down approximation (CSDA) range of 1 m, 10 m, 100 m and 1000 m). The muon’s decay length (660 × γ m) is extended depending on its relativistic level that is measured by the Lorentz factor γ. These relativistic muons in water travel faster than light in water. For example, the speed of muon that can reach the 500 m deep seafloor is more than 0.9999999*c*. In conjunction with this universality and their relativistic; hence penetrative nature, the technique called muography has been widely applied to visualizing the internal structure of gigantic objects including volcanoes and historical heritage in Africa^20,21^, the Americas^22–24^, Asia^25–31^, and Europe^32–38^.

Likewise, by utilizing this universality and relativistic nature, cosmic muons have a potential to be used for positioning the receiver detector located underwater or underground three dimensionally with a great accuracy within the coordinate defined by the reference detectors. Since cosmic muons always precipitate from the upper hemisphere, multiple particle detectors (reference detectors) located above a receiver detector provide the times of flight between these reference detectors and a receiver detector, and this information can be simply converted to the distances between these detectors by multiplying the speed of light in a vacuum.

Distance ranging by measuring the time of flight (TOF) of muons has been applied to the adjustment of detector alignments^39, 40^. There also have been some attempts to monitor the stability of historical buildings^41^. However, the range has been limited thus far: typically, from a few meters to the 10-m scale. There has not yet been an application, demonstration or proposal for using the TOF of muons for three-dimensional and kilometer-scale-long-range positioning that could be applied to monitoring the offshore seafloor deformation. In this work, a laboratory-scaled muometric positioning system (μPS) was developed, and kilometer-scaled-three-dimensional positioning was demonstrated by combining lab-scale experiments and the digital circuit-driven emulation of a TOF of muons in a long time range up to 5 microseconds (equivalent to a traveling distance of 1.5 km for relativistic muons). Moreover, in order to evaluate the technological feasibility for using this TOF technique at a deep seafloor site, Monte-Carlo simulations were performed to evaluate the scattering effect of relativistic muons for positioning under the sea. The results indicated that the plate-tectonics-driven seafloor motion and magma-driven seamount deformation can be detected with μPS.

## Results

### Development and demonstration of the short-range μPS

**Figure 1:**
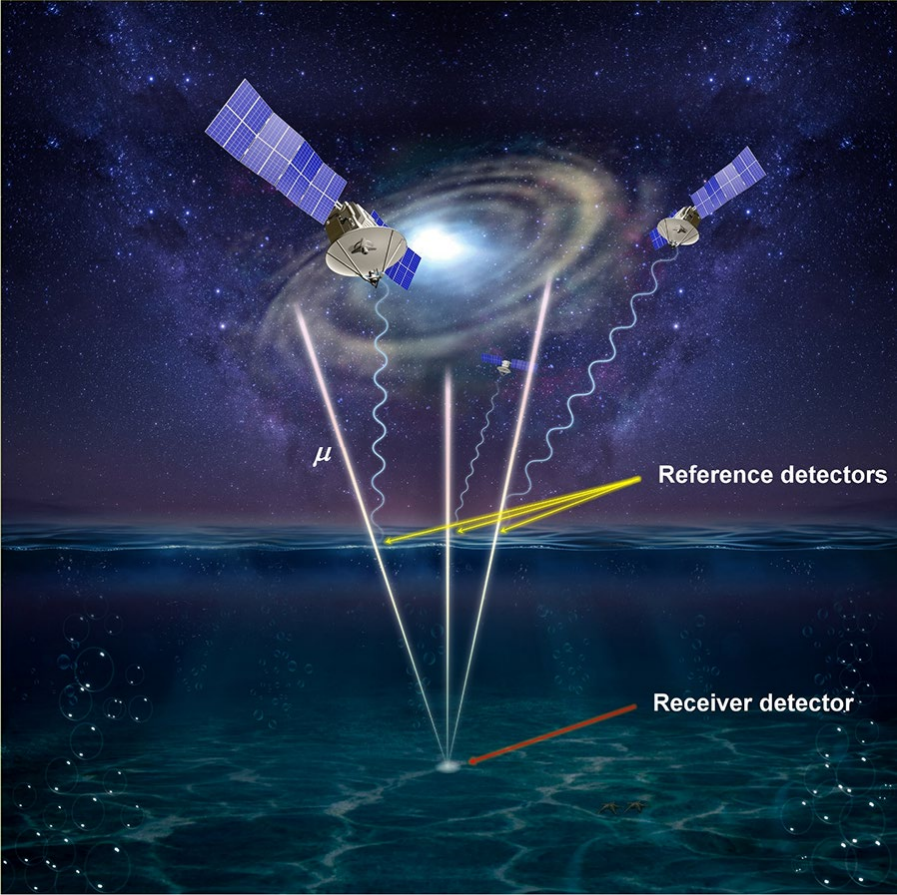
Conceptual view of the muometric positioning system (μPS). The symbol μ indicates a muon. The copyright of this image is owned by HKMT.

The μPS consists of multiple reference detectors and one receiver detector (Fig. 1). These reference detectors define the coordinate of the entire system and the receiver detector defines the relative position within this coordinate by using the following relationship:

1 $$L_{i}^{2} = \, \left( {x_{i} - x_{p} } \right)^{2} + \left( {y_{i} - y_{p} } \right)^{2} + \left( {z_{i} - z_{p} } \right)^{2}$$

where *L*_*i*_ is the geometrical distance between the *i*th reference detector located at (*x*_*i*_, *y*_*i*_, *z*_*i*_) and the receiver detector located at (*x*_p_, *y*_p_, *z*_p_). The location of the reference detectors could be defined by using the global positioning system.

**Figure 2:**
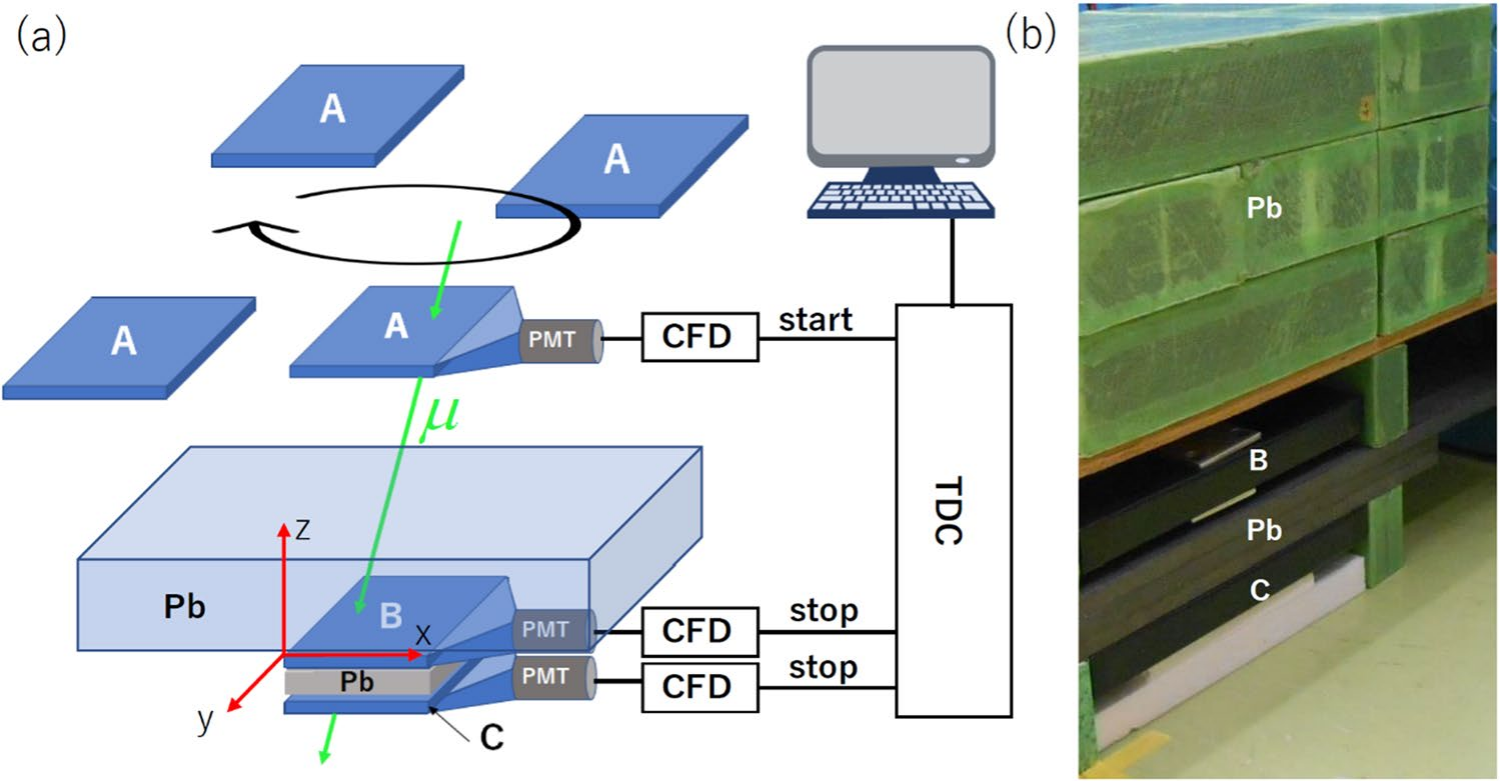
Experimental setup. The symbol A, B, C, Pb, PMT, CFD and TDC respectively indicate the reference, receiver, checking detectors, the lead block, the photomultiplier tube, the constant fraction discriminator and the time to digital converter (**a**). The close view of the receiver and checking detectors is shown in the photograph (**b**). The reference detector was moved for calculating the position of the receiver detector.

The μPS setup developed for the current experiment is summarized in Fig. 2. Here the scintillators are labeled Detector A, B, and C from the upward to the downward along the cosmic muon's traveling direction. Detector A is the reference detector and randomly moved for calculating the position of B. Detector B located at the origin of the coordinate (*x*_p_, *y*_p_, *z*_p_) = (0, 0, 0) is the receiver detector (Fig. 2a), and the checking detector C was used for selecting only the relativistic muons. Between Detectors A and B, a 15-cm thick lead block (equivalent to 1.8-m water) was placed, and a 3-cm thick lead block was inserted between B and C (Fig. 2b). This lead block was used for rejecting non-relativistic slow muons (< 0.9c)^42^. The time between Detectors A and B was measured only when A, B and C generates the signal within the given time window so that the non-relativistic muons are removed (See “Methods” Section).

**Figure 3:**
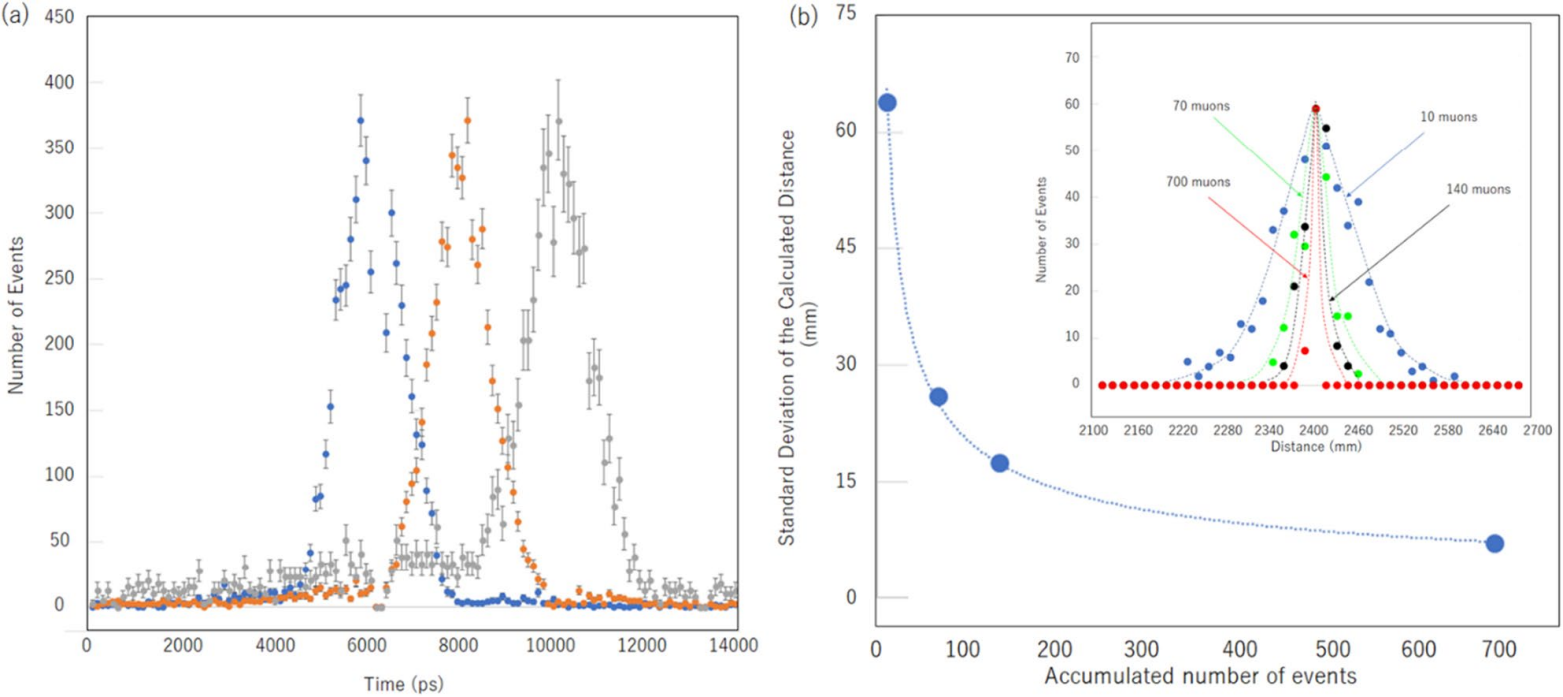
Time spectra measured between the reference and receiver detectors. They were measured for different given distances of 1.8 m (blue circles), 2.4 m (orange circles), and 3.0 m (gray circles) (**a**). The error bars show the standard deviation. Accuracy in the distance calculation as a function of the number of events (**b**). The inset shows the TOF spectra for different accumulation numbers: 10 muons, 70 muons, 140 muons, and 700 muons. The horizontal axis was converted to the distance by multiplying the speed of light in a vacuum.

The overall jitter coming from the scintillator including the size (20 × 20 cm^2^) and rise time effect, PMT, CFD, and TDC were ± 1 ns (corresponding to an error of ± 30 cm in positioning) as shown in the TOF spectra (Fig. 3a), being independent from the distance between the reference and receiver detectors (1.8 m, 2.4 m, 3.0 m). As was expected, the peaks of the spectra were respectively located at 6, 8, and 10 ns. The plots for the A-B distance of 3.0 m shows a higher background level in the shorter time range because the accidental coincidence due to the shower particles increased as the A-B angle was slanted (45°). The small tails extended towards the longer time direction are due to the semi-relativistic muons (> 0.9c) that could not be cutoff by the lead block between Detectors B and C, but its contribution is negligible. By averaging over multiple muon tracks, these spectra are naturally sharpened (Fig. 3 inset), with the standard deviations of ± 6 cm, ± 3 cm, ± 1.5 cm, and ± 7 mm after averaging over 10, 70, 140, and 700 tracks, respectively (Fig. 3b).

**Figure 4:**
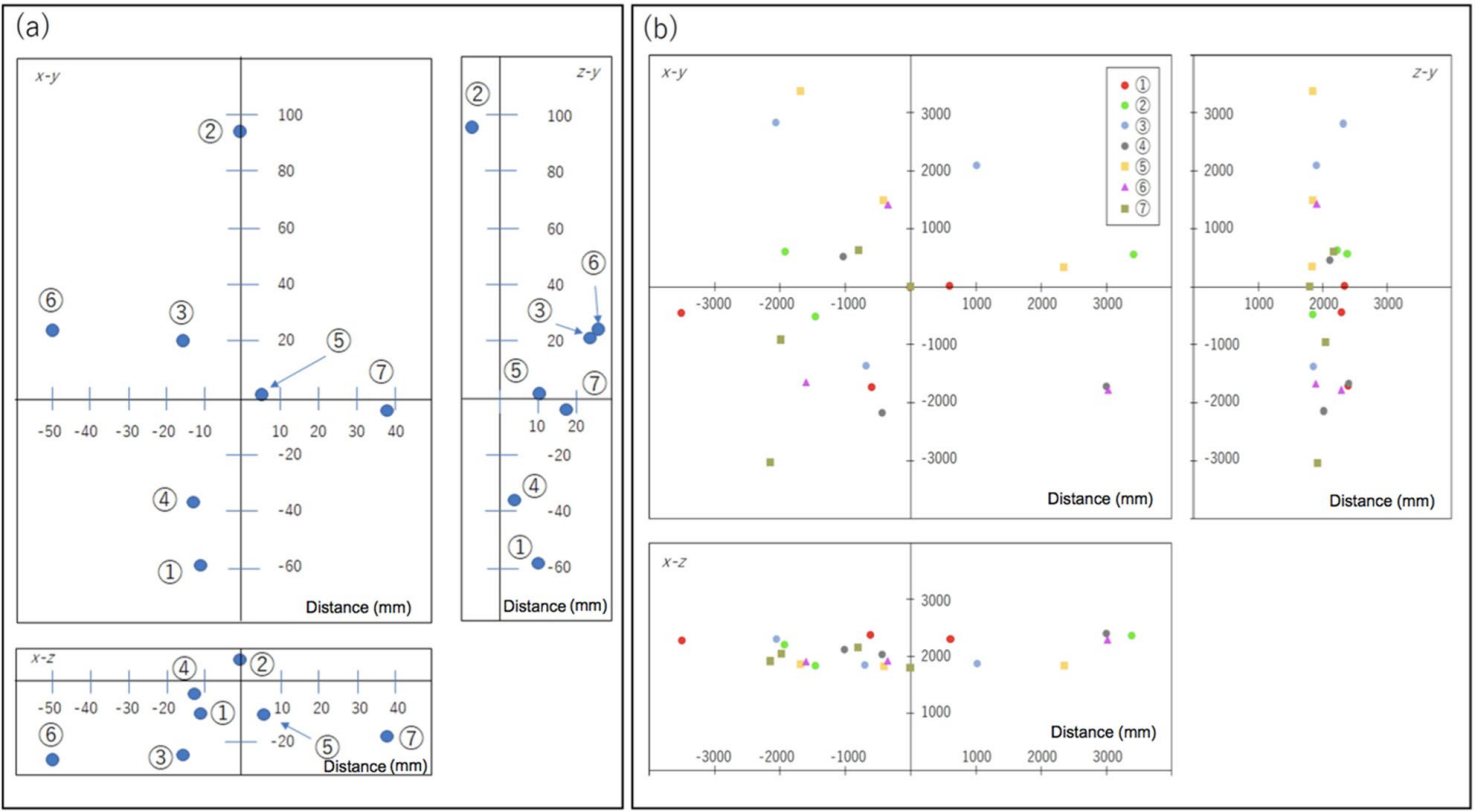
Positioning results. The determined positions of the receiver detectors are indicated by blue filled circles that are shown on the *x–y*, *z-y* and *x–z* planes (**a**). The circled numbers indicate the run numbers. The positions of the reference detectors used for positioning receiver detectors are also shown (**b**). The colors of the filled circles identify the run numbers. The origin of the coordinate in these plots was defined by the position of one of the receiver detector’s corners as shown in Fig. 1. The numbers associated with the vertical and horizontal axes indicate the distance from the origin of the coordinate in units of mm.

Figure 4a shows the positioning result. For each positioning process, four randomly generated positions (Fig. 4b) were used, but one of them was fixed at a position right above the receiver detector ((*x, y, z*) = (0 cm, 0 cm, 180 cm)) to confirm the positioning accuracy under ideal conditions. The number of tracks used for averaging the TOF spectrum was 10^2^ for each process. The same process was repeated 7 times for confirmation of reproducibility as is indicated by the run numbers in Fig. 4. The resultant positioning errors (standard deviation) associated with the *x*, *y*, and *z* directions over the entire processes were respectively 24.5 mm, 45.8 mm, 11.1 mm. As was expected, the vertical position was well defined, indicating that the currently developed μPS can attain an accuracy of 1 cm with an ideal geometrical configuration of the reference detectors.

### Emulation of the long-range μPS

**Figure 5:**
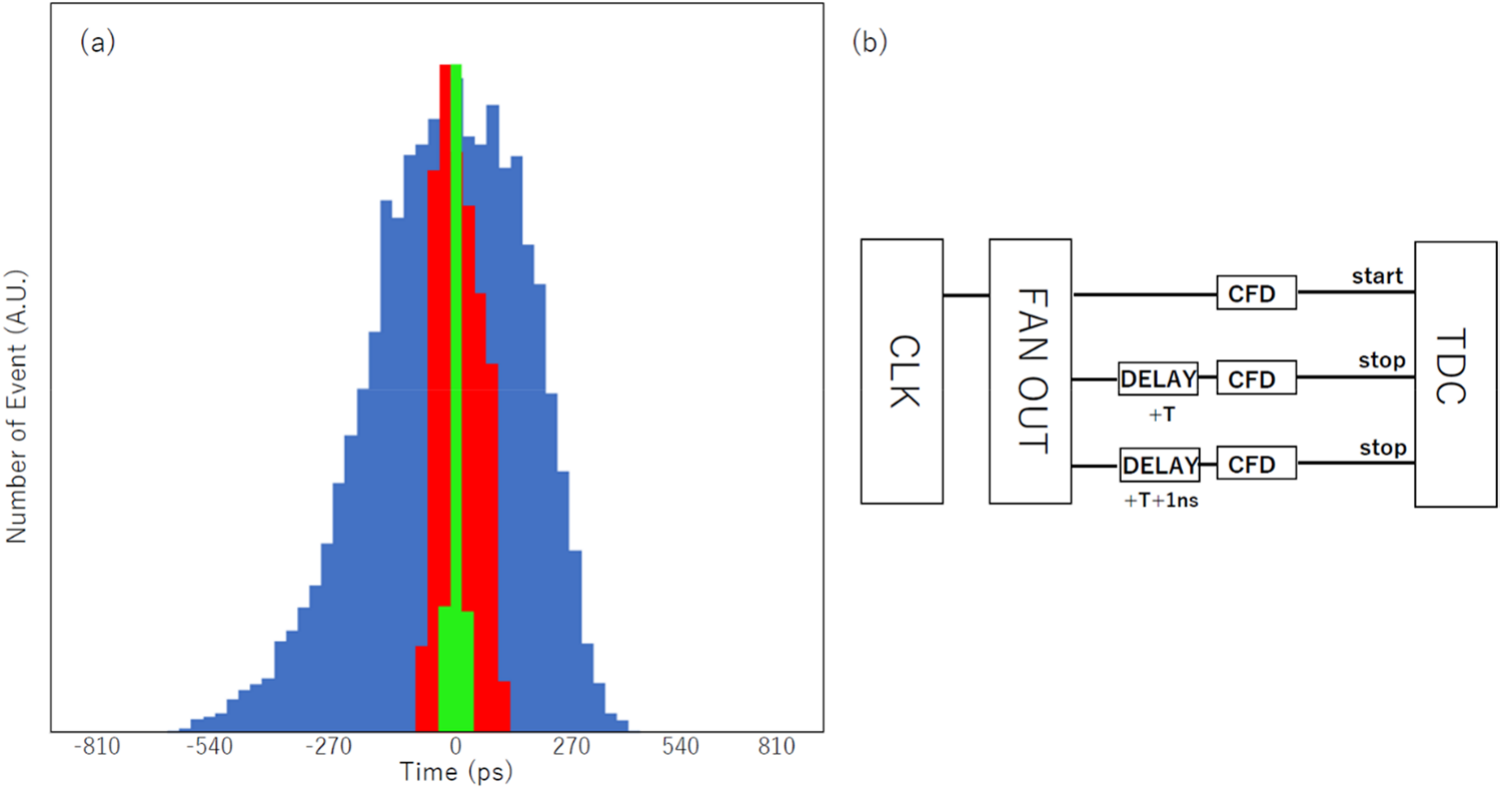
Time spectra measured for different delay times. Each bin width is 27 ps. The spectra measured for the delay times of 50 ns (green), 500 ns (red), and 5 μs (blue) are plotted (**a**). A block diagram of the electronics used for this measurement is also shown (**b**). The symbol T indicates the delay time.

This result can be simply scaled up to longer baseline positioning by extending the distance between the reference and receiver detectors, but it is difficult for us to actually implement hectometric to kilometric-scale experiments in the laboratory. However, it is reasonable for us to assume the detector-associated jitter (a simple compilation of scintillator, PMT and CFD jitters) is independent from the A-B distance. Therefore, the evaluation of the timing errors of the current μPS with artificially delayed signals will emulate a larger scale measurement (See “Methods” section). Figure 5 shows the resultant time spectra measured for the different delay times of 50 ns, 500 ns, and 5 μs that are respectively equivalent to the A-B distances of 15 m, 150 m, and 1.5 km. As can be seen in this figure, the level of the jitter increases as the measurement time range becomes longer, and they were 50 ps, 200 ps, and 700 ps (standard deviation) for the time intervals of 50 ns, 500 ns, and 5 μs, respectively. These levels of jitters are still smaller than the detector-associated jitter, and we now can conclude that our current μPS setup requires ~ 10^3^ muon events to determine the A-B distance at a 1-cm level accuracy regardless of the distance between the reference and receiver detectors.

### Monte-Carlo simulations of the ocean-based μPS

**Figure 6:**
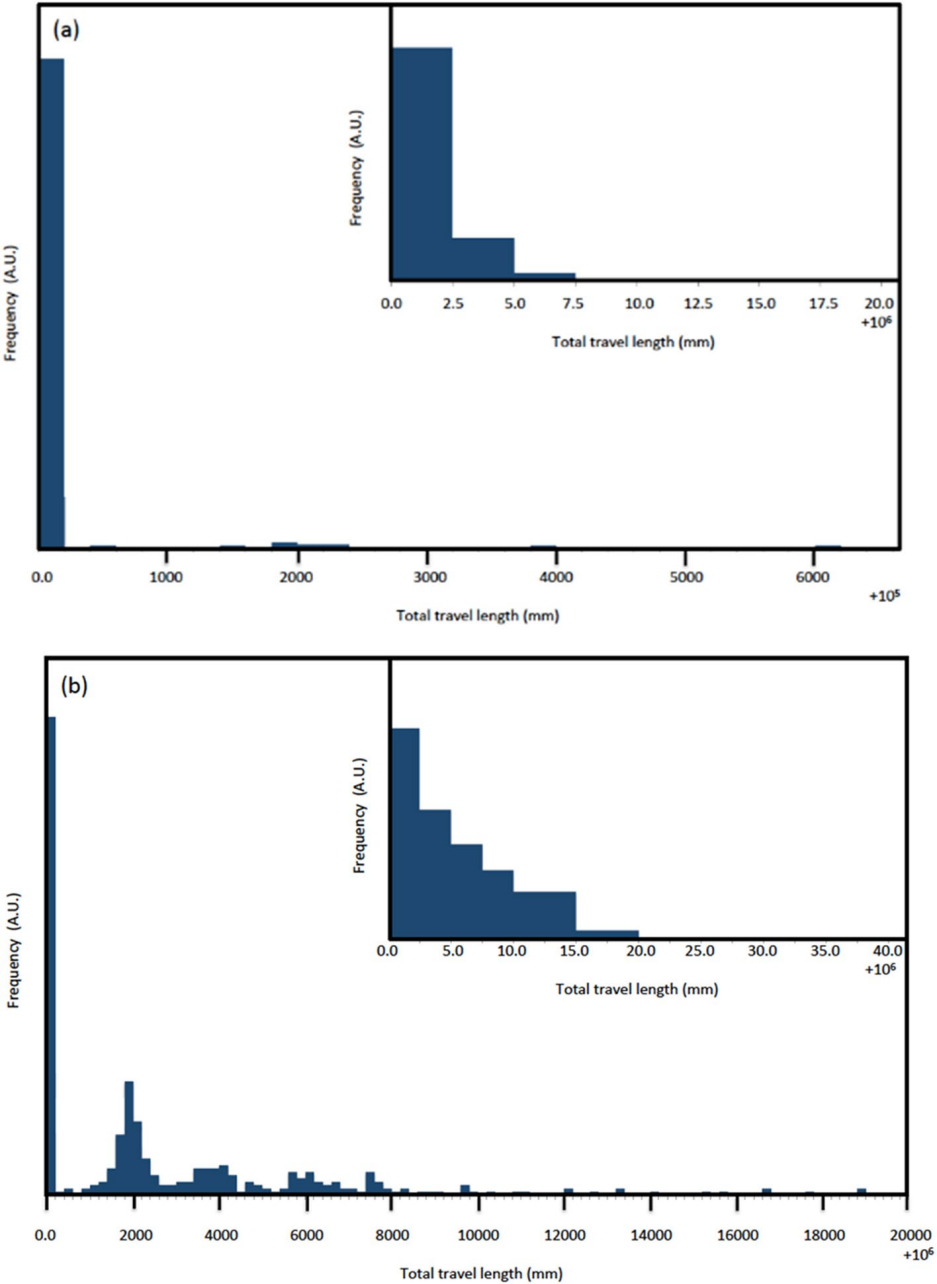
Distribution of the muon’s total travel lengths. The receiver detector is located at different depths of 100 m (**a**) and 1 km (**b**). The plots are shown as an excess from the linear trajectory. The inset shows the detailed presentation of the distribution near the primary peak.

As long as the energy cutoff guarantees the sufficiently relativistic muons at the receiver detector, the reference-receiver distance can be simply calculated by *ct*_AB_, where *t*_AB_ is the TOF measured between the reference and receiver detectors. However, this calculation only holds with an assumption of the linear muon trajectories. Figure 6 shows the results of the Monte Carlo simulations to estimate the muon’s total travel length in water for different thicknesses (100 m and 1000 m) with an energy cutoff of 10 GeV at the receiver detector (See “Methods” Section). The standard deviations of the primary distributions in Fig. 6 (inset) were respectively 1.8 and 4.2 mm for 100 m and 1000 m, indicating most of the muon trajectories are linear.

The secondary radiative peaks are negligible for a thickness of 100 m, but more serious for 1 km. The radiative processes are characterized by small cross sections but large scattering angles, and thus it generates the additional travel length. However, these radiative peaks are clearly separated from the primary peak, and can be removed in the practical analysis.

## Discussion

Now we can draw a picture of how to apply the μPS to positioning the seafloor. The ocean-based μPS could be deployed as a combination with the GPS at sea level (Fig. 1). With this scheme, the locations of the reference detectors will be determined by the GPS and their positioning accuracy depends on the GPS quality. For example, the reference and receiver detectors could be respectively equipped with the GPS buoys and the anchor. The typical size of the GPS buoy is 5 m in diameter fixed by a 30-ton anchor, having a sufficient capacity to load particle detectors. In this case, the natural motion of the buoy collects the data for different locations above the receiver detector.

By providing additional directional information, the ocean-based μPS will advance our understanding about the tectonic or magmatic driven seafloor motion because most of the conventional techniques can only guarantee their accuracy in either vertical or horizontal direction. Here we consider the following three cases as an example to estimate the size of the detector and the measurement time required to attain the same level accuracy they achieved in their specific directions: (A) shallow vertical seafloor displacement caused by Campi Flegrei volcanic activities reported by Chierici et al.^5^, (B) deep seafloor vertical deformation caused by magmatic activities at Axial Seamount reported by Chadwick Jr. et al.^3^, and (C) plate tectonics driven deep seafloor horizontal motion reported by Gagnon et al.^9^. The measurement depth and deformation rates for (A), (B) and (C) are respectively 100 m, 1500 m, and 2000 m; and 3 cm/100 days, 2 m/10 years and 6 cm/year.

Since the solid angle to collect muons is approximately *S*^2^*L*^−2^ sr, the time required for collecting *N*_μ_ muons will be *N*μ*L*^2^*S*^−2^*I*_μ_^−1^, where *S* and *I*_μ_ are respectively the active area of the detector and the muon flux. By using this relationship and the current experimental results, the optimal detector size (and the measurement time) could be calculated as follows: 1-m^2^ detectors to attain a few cm accuracy within 100 days for Case (A), 4-m^2^ detectors to attain 10 cm accuracy within 100 days for Case (B), and 20-m^2^ detectors to attain 5 cm accuracy within 1 year for Case (C) by considering the muon flux (*I*_μ_) at different depths^19^.

**Figure 7:**
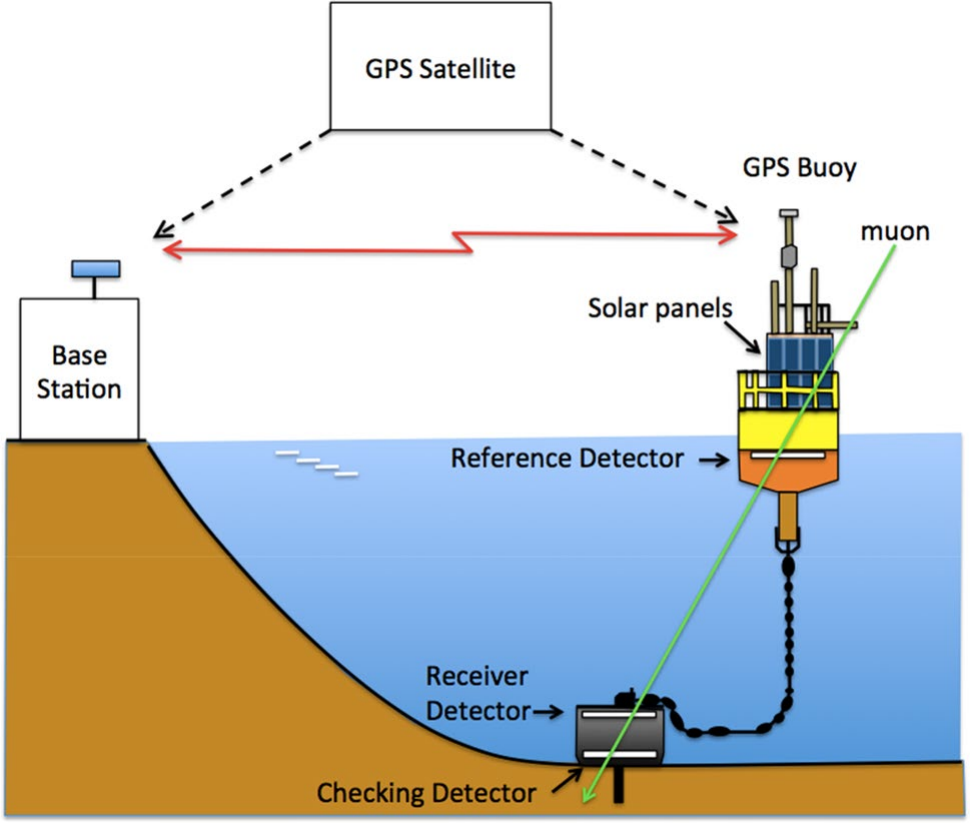
Deployment of μPS to be installed on the Hitachi-Zosen GPS buoy. The copyright of this image is owned by HKMT.

In conclusion, we successfully developed the μPS methodology in this current work. Our laboratory-based testing demonstrated that the system could position the receiver detector at a cm level accuracy. In conjunction with the Monte-Carlo simulations of the muon propagation through water, it was indicated that this system could be applied to monitoring of the seafloor displacement. Figure 7 shows a possible scheme to deploy μPS for seafloor positioning. In this work, the deployment of μPS to be installed on the Hitachi-Zosen GPS buoy^43^ was proposed. Specifications of this GPS buoy are summarized in Table 1. This GPS buoy was developed for monitoring the offshore tsunami propagation. Large-sized solar panels are attached in every azimuthal direction to the buoy, and it was designed to tolerate against harsh marine conditions for long-term stable monitoring. 18 of the same models of this GPS buoy are currently under operation in Japan.

**Table 1:** Specifications of the Hitachi-Zosen GPS buoy.

Buoy diameter (m)	4.5
Buoy height (m)	17.2
Buoy weight (tons)	38.0
Anchor weight (tons)	27.0
Onboard sensors	Water flow velocimeter
	Water current meter
	Water temperature gauge
	Air temperature indicator
	Anemometer
	Barometer
	GPS antenna
	Radio antenna

These GPS buoys are operated 20 km offshore and communicate with a land-based GPS station at the coast to receive real-time-kinematic-GPS (RTK-GPS) correction data. A steel chain connects this 38-ton buoy to a 27-ton anchor that is embedded into the seafloor. The reference detector that could be placed inside this buoy would measure 4.5 m in diameter (16 m^2^ in area); additionally, the receiver detector and the checking detector will be respectively embedded into the top and the bottom part of this anchor. This massive anchor also acts as a radiation shield for non-relativistic muons. The receiver and checking detectors would be powered through a cable attached to the chain. Discriminated muon pulses detected by the receiver detector will be transferred to the buoy through a signal cable attached to the chain to derive the time of flight between the reference detector and receiver detector. Due to the slewing motion of the buoy, the RTK-GPS device will record the time and the multiple positions of the reference detector with an accuracy of a few centimeters. The TOF between the reference detector and receiver detector will be combined with the information about these multiple positions and times of the reference detector in order to position the anchor of the buoy in the same way as described in the “Results” section. By fully utilizing the space available inside the buoy and anchor, the aforementioned cases from (A) to (C) can be investigated.

Regarding the communication between the buoy and anchor, the synchronization of the reference, receiver and checking detectors will be a major factor to be considered. Seasonal temperature variations of seawater (for example, in the Pacific Ocean at 160° E 30° N, they are typically ± 5 °C) will cause errors associated with fluctuations in the length of the cables. For example, the linear expansion rate of copper is 1.7 mm/K for a 100 m cable. This expansion rate will lead to seasonal variations in the detection time at the receiver detector located at a long distance (up to 1.5 km) to be up to ± 400 ps. However, in many cases, these variations only come from the surface layer of the sea (seasonal thermocline), and the Hitachi-Zosen GPS is already equipped with a water temperature gauge, which can monitor surrounding temporal variations. For example, the depth of the seasonal thermocline in the Pacific Ocean at 160° E 30° N is ~ 50 m, and there are almost no seasonal variations in temperature at places deeper than this. Furthermore, signal cables made of materials with smaller thermal expansion rate, e.g., tungsten (0.4 mm/K for a 100 m cable), the seasonal variations associated with thermal expansion of the signal cables will be further suppressed at an almost negligible level (± 6 ps). Another possibility to affect the cable length would be mechanical expansion by the strong sea current near the seafloor. Therefore, the signal cables have to be inserted into the mechanically robust flexible tube.

In conclusion, the effect in positioning from temperature, salinity or any possible conditional changes of seawater can be monitored and minimized in the μPS. Moreover, this μPS technique utilizes passive secondary cosmic rays without the necessity of generating an artificial probe source, and thus there is no adverse effect to marine bio-activities. This technique is also adaptable to underground positioning as long as the position of the reference detector is known.

## Materials and methods

### Laboratory-based positioning experiment

The time resolution of the time to digital converter (TDC) used for the current experiment (Technoland N-TDC 019) was 27 ps (equivalent to a distance of 8 mm at the speed of light), and the measurable time range was 5 microseconds (equivalent to a distance of 1.5 km at the speed of light). The photons generated in the plastic scintillator (ELJEN 200) with a size of *D*^2^ (= 20 × 20 cm^2^) and with a thickness of 2 cm were guided through an acryl light guide, read out by the photomultiplier tube (PMT) (Hamamatsu R7724) and discriminated by using the constant fraction discriminator (CFD) (KAIZU KN381). The voltage applied to the PMT was 1600 V and the pulse height was attenuated with the variable attenuator (Technoland N-TM 224a) so that the maximum pulse height would be lower than − 800 mV. The signal discrimination level was − 50 mV.

### Long time range measurement

The long TOF signals were emulated using a combination of the clock generator (Technoland N-RY 024) and a variable delay (Technoland N-TM 225a). The delay time was adjusted by monitoring the delay signals with an oscilloscope. The signals from the reference and receiver detectors were respectively emulated by the direct output and delayed output from the clock generator. The signal prepared for the receiver detector were further delayed by 1 ns for the checking detector. All of the signals were discriminated by the CFD (KAIZU KN381) before being fed into the TDC (Technoland N-TDC 019).

### Simulations

In the current simulation, muons were vertically injected to the reference detector, water layer and receiver detector according to the cosmic muon energy spectrum at sea level^19^, and only muons that successfully reached the checking detector after passing through the 5-m thick lead (muons with energies roughly above 10 GeV at the receiver detector) were considered. One layer was defined as one assembly volume that consisted of a sequence of the first detector (reference detector made of a plastic scintillator), first absorber (water), second detector (receiver detector made of a plastic scintillator), second absorber (lead block), and third detector (checking detector made of a plastic scintillator). The water thickness was varied from 100 m to 1000 m, but the thicknesses of other volumes (2 cm for detectors and 5 m for lead) were fixed. The sizes of the detectors and lead block were all fixed to be 10^4^ m^2^ (100 × 100 m^2^). In order to save the computing time, only the muons with energies above 20 GeV and 200 GeV were injected to 100-m thick water and 1000-m thick water, respectively. The CSDA ranges of these muons in water are respectively 82 m and 680 m. Salinity of seawater was not considered in the current simulation, but the density of water, ρ, was fixed to be 1.02 g cm^−3^. The propagation of the 100,000 injected muons through the medium were simulated by using the Geant4 Monte Carlo simulation toolkit^44^ which incorporates the muon's multiple coulomb scattering, decay, and energy loss processes via ionization, Bremsstrahlung, direct pair production, and photonuclear interactions. The muon’s energy deposition and track length were recorded at the depth of the receiver detector.
